# Relationship between Stage of Chronic Kidney Disease and Sarcopenia in Korean Aged 40 Years and Older Using the Korea National Health and Nutrition Examination Surveys (KNHANES IV-2, 3, and V-1, 2), 2008–2011

**DOI:** 10.1371/journal.pone.0130740

**Published:** 2015-06-17

**Authors:** Sung Jin Moon, Tae Ho Kim, Soo Young Yoon, Jae Ho Chung, Hee-Jin Hwang

**Affiliations:** 1 Department of Internal Medicine, International St. Mary’s Hospital, Catholic Kwandong University, Incheon, Korea; 2 Department of Internal Medicine, Seoul Medical Center, Seoul, Korea; 3 Department of Family Medicine, International St. Mary’s Hospital, Catholic Kwandong University, Incheon, Korea; UNIFESP Federal University of São Paulo, BRAZIL

## Abstract

**Background:**

Protein-energy wasting is common in patients with end-stage kidney disease. However, few studies have examined the relationship between early stages of chronic kidney disease (CKD) and sarcopenia.

**Methods:**

We conducted a cross-sectional study based on data in the Korea National Health and Nutrition Examination Survey, 2008–2011. In total, 11,625 subjects aged 40 years or older who underwent dual-energy X-ray absorptiometry were analyzed. Sarcopenia was defined based on values of appendicular skeletal muscle mass as a percentage of body weight (ASM/Wt) two standard deviations below the gender-specific mean for young adults. Estimated glomerular filtration rates (eGFR) were calculated using the CKD-EPI equation.

**Results:**

Mean age, body mass index (BMI), and HOMA-IR were higher and caloric intake, physical activity, and vitamin D level were lower in the sarcopenia groups in both men and women. As the stage of CKD increased, the prevalence of sarcopenia increased, even in the early stages of CKD (normal and CKD1, 2, and 3-5: 2.6%, 5.6%, and 18.1% in men and 5.3%, 7.1%, and 12.6% in women, respectively; p < 0.001). In addition, a correlation analysis showed that GFR and ASM/Wt had significant correlations in both men and women. Logistic regression analyses, after adjusting for age, BMI, caloric intake, log(physical activity), vitamin D level, and log(HOMA-IR), showed that the odds ratio for sarcopenia with respect to CKD 3–5 was 1.93 (95% CI = 1.02–3.68) in men but was not statistically significant in women.

**Conclusions:**

The prevalence of sarcopenia was higher in elderly Korean patients with even mildly reduced kidney function. Stage of CKD was associated with an increased prevalence of sarcopenia in men but not women. Thus, we should evaluate the risk of sarcopenia and work to prevent it, even in patients with early CKD.

## Introduction

Loss of muscle mass, especially skeletal muscle mass, is an important age-related change. The term ‘sarcopenia’ is used to describe progressive muscle mass loss associated with aging [1]. The reported prevalence of sarcopenia ranges from 7% to 24% due to the lack of a generally accepted definition and heterogeneity within study populations [2]. The European Working Group on Sarcopenia in Older People (EWGSOP) published a report titled “Sarcopenia: European consensus on definition and diagnosis” in 2010 [3]. Loss of lean body mass leads to frequent falls, osteoporosis, and related complications [4]. Sarcopenia is associated with the risk of adverse outcomes such as physical disability, poor quality of life, and death [5].

Recently, the prevalence of chronic kidney disease (CKD) has been increasing due to the proportion of individuals with obesity, diabetes, and hypertension [6,7]. In CKD patients, the loss of muscle mass is more severe and occurs earlier than that in their peers [8]. In addition, sarcopenia is more commonly observed in patients with end-stage renal disease. Foley’s 2007 report “Kidney function and sarcopenia in the United States general population: NHANES III” is the most well-known study about the relationship between kidney function and sarcopenia [9]. Since that report, however, there have been few studies on the relationship between muscle mass and mild to moderate decreased kidney function. Most studies on the relationship between muscle mass and kidney have been conducted in Western countries and have focused on end-stage renal disease subjects undergoing hemodialysis. Therefore, we investigated the relationship between early CKD and sarcopenia in an aged Korean population using data from the Korea National Health and Nutrition Examination Survey (KNHANES), a cross-sectional, nationally representative survey conducted by the Division of Chronic Disease Surveillance, Korea Centers for Disease Control and Prevention.

## Methods

### Study participants and database

We used data acquired in the second and third years (2008–2009) of the KNHANES IV and the first and second years (2010–2011) of the KNHANES V. The KNHANES has been conducted periodically since 1998 to assess the health and nutritional status of the non-institutionalized civilian population of South Korea. Annually, 10,000–12,000 individuals from 4,600 households are selected to represent Koreans aged 18 years or older using a multi-stage clustered and stratified random sampling method based on national census data.

Of the 35,722 participants, we excluded subjects <40 years old (n = 15,086), those who did not receive an examination with dual-energy X-ray absorptiometry (n = 8,238), those with any malignancies, hepatitis, and autoimmune diseases (n = 629), and those with no serum creatinine data (n = 144). Finally, the analysis included 11,625 subjects aged ≥ 40 years who underwent anthropometric measurements, laboratory tests, and dual-energy X-ray absorptiometry. Height, weight, and blood pressure were measured. Body mass index (BMI) was calculated by dividing weight (kg) by height squared (m^2^). Blood samples were obtained in the morning following an overnight fast. The homeostasis model assessment estimate of insulin resistance (HOMA-IR) was calculated as fasting insulin (mIU/mL) × fasting glucose (mg/dL)/405. Physical activity was ascertained by asking participants how often they engaged in exercise each week using the Korean version of the International Physical Activity Questionnaire (IPAQ). Using the Ainsworth et al. Compendium, an average MET score was derived for each type of activity. The following values were used for the analysis of IPAQ data: Walking = 3.3 METs, Moderate physical activity (PA) = 4.0 METs, and Vigorous PA = 8.0 METs. Using these values, four continuous scores were defined as Walking MET-minutes/week = 3.3 x walking minutes x walking days, Moderate MET-minutes/week = 4.0 x moderate-intensity activity minutes x moderate days, Vigorous MET-minutes/week = 8.0 x vigorous-intensity activity minutes x vigorous-intensity days, and total physical activity MET-minutes/week = sum of Walking + Moderate + Vigorous MET-minutes/weeks scores.

### Definition of sarcopenia

Whole and regional body compositions were measured using whole-body dual-energy X-ray absorptiometry (DXA; Discovery-W, Hologic, Inc., Waltham, MA, USA). Appendicular skeletal muscle mass (ASM) was calculated as the sum of skeletal muscles in the arms and legs, assuming that all non-fat and non-bone tissue was skeletal muscle. We defined sarcopenia using ASM as a percentage of body weight (ASM/Wt), modified from a previous study [10]. ASM/Wt values that were two standard deviations (SD) below the gender-specific mean for the younger reference group (6,199 subjects, aged 19–39) in the KNHANES IV-V were used to identify sarcopenia. The cut-off ASM/Wt value for sarcopenia was 27.24% in men and 21.31% in women.

### Stages of CKD

Estimated glomerular filtration rate (eGFR), expressed in mL/min/1.73 m^2^, was calculated using the CKD-EPI equation: for females with a serum creatinine level ≤0.7 mg/dL, GFR = 144 × (serum creatinine/0.7)^-0.329^ × (0.993)^Age^; for females with a serum creatinine level >0.7 mg/dL, GFR = 144 × (serum creatinine/0.7)^-1.209^ × (0.993)^Age^; for males with a serum creatinine level ≤0.9 mg/dL, GFR = 141 × (serum creatinine/0.9)^-0.411^ × (0.993)^Age^; for males with a serum creatinine level >0.9 mg/dL, GFR = 141 × (serum creatinine/0.9)^-1.209^ × (0.993)^Age^ [11]. GFR was classified according to the system suggested in the Kidney Disease Outcome Quality Initiative of 2002 [12]. Due to the small sample sizes, subjects with stage 3, 4, and 5 kidney function were combined into one group, leaving the following GFR groups for comparative analyses: normal and CKD 1, ≥90; CKD 2, 60–89.9; and CKD 3–5, <60 mL/min/1.73 m^2^.

### Ethical issues

All participants in the KNHANES IV and V survey signed an informed consent form. As this was a cross-sectional study that used and analyzed data from the KNHANES IV and V survey (http://knhanes.cdc.go.kr/knhanes/), ethical approval was not required.

### Statistical analysis

Statistical analysis was performed using SPSS for Windows ver. 15.0 (SPSS, Inc., Chicago, IL, USA). Continuous variables are expressed as mean ± SD and were compared using Student’s t-test. Categorical variables are expressed as proportion and were compared using the Chi-square test. All analyses were performed separately for men and women. The relationships between ASM percentage and eGFR were examined using Spearman correlation analysis. To identify the independent factors associated with sarcopenia, multivariate logistic regression analysis was performed after adjustments for age, BMI, caloric intake, physical activity, vitamin D, HOMA-IR, and stage of chronic kidney disease. Due to skewed distributions, physical activity and HOMA-IR were log-transformed before multivariate analysis. P-values less than 0.05 were considered to be statistically significant.

## Results

Anthropometric characteristics of the subjects are provided in Table 1. The mean age was higher in patients with sarcopenia. Weight, BMI, waist circumference, and total fat mass were higher in participants with sarcopenia, but total muscle mass was lower. Family income and educational status were lower in the sarcopenia group, and the prevalence of comorbid disease such as diabetes mellitus, hypertension, and cardiovascular disease was higher. Calorie, protein, and fat intakes were lower in the sarcopenia group, as was physical activity. WBC and triglyceride, fasting glucose, and HOMA-IR levels were higher in both genders with sarcopenia. However, total cholesterol and serum ferritin were higher in the sarcopenia group in women, and HDL cholesterol was significantly lower in the sarcopenia group in men. In addition, vitamin D was lower in the sarcopenia group, and ALP and intact parathyroid hormone were higher.

**Table 1:** Clinical characteristics of Koreans aged 40 years or older according to sarcopenia status.

	Men			Women		
Variables	Without sarcopenia (*n* = 4831)	With sarcopenia (*n* = 239)	p-value	Without sarcopenia (*n* = 6148)	With sarcopenia (*n* = 407)	p-value
**Age (years)**	57.1±11.3	64.7±11.4	<0.001	57.4±11.5	62.9±11.1	<0.001
** 40–59**	2840(58.8)	66(27.6)	<0.001	3590(58.4)	164(40.3)	<0.001
** 60–70**	1174(24.3)	74(31.0)		1444(23.5)	104(25.6)	
** ≥ 70**	817(16.9)	99(41.4)		1114(18.1)	139(34.2)	
**Height (m)**	1.68±0.06	1.65±0.06	<0.001	1.55±0.06	1.52±0.05	<0.001
**Weight (kg)**	67.4±9.9	72.6±10.8	<0.001	57.1±8.5	63.3±9.6	<0.001
**BMI (kg/m^2^)**	23.8±2.9	26.5±3.1	<0.001	23.8±3.1	27.4±3.6	<0.001
**Waist circumference (cm)**	84.8±8.3	94.0±8.3	<0.001	80.4±9.0	90.1±9.6	<0.001
**Total fat mass (kg)**	14.8±4.6	22.7±4.6	<0.001	19.0±4.9	27.0±5.3	<0.001
**Total muscle mass (kg)**	49.5±6.4	46.7±6.6	<0.001	35.8±4.4	34.0±4.6	<0.001
**Family income (low)**	1023 (21.4)	87 (37.2)	<0.001	1663(27.4)	143(35.5)	<0.001
**Education (< middle)**	2061 (42.9)	126 (53.2)	0.02	3736(61.2)	316(78.4)	<0.001
**Diabetes mellitus**	553 (11.4)	64 (26.8)	<0.001	580(9.4)	71(17.4)	<0.001
**Hypertension**	1393 (28.8)	139 (58.2)	<0.001	1756(28.6)	206(50.6)	<0.001
**Cardiovascular disease**	200 (4.1)	28 (11.7)	<0.001	178(2.9)	24(5.9)	<0.001
**Caloric intake (kcal/day)**	2231±853	1873±681	<0.001	1613±610	1456±523	<0.001
**Protein intake (g/day)**	78.9±39.2	64.0±29.3	<0.001	55.4±27.4	49.5±29.8	<0.001
**Fat intake (g/day)**	38.7±29.0	31.1±23.8	<0.001	26.1±20.2	23.3±18.2	0.008
**Physical activity (MET-min/weeks)**	3446±4771	2349±3353	0.002	2656±4213	2157±3755	0.020
**WBC (/mm^3^)**	6.46±1.72	7.30±2.23	<0.001	5.68±1.55	6.45±1.79	<0.001
**Hemoglobin (g/dL)**	15.0±1.2	14.9±1.4	0.232	13.0±1.1	13.2±1.1	<0.001
**Total cholesterol (mg/dL)**	189±36	189±40	0.896	196±36	205±39	<0.001
**Triglyceride (mg/dL)**	165±140	185±142	0.031	128±85	144±79	<0.001
**HDL cholesterol (mg/dL)**	49±12	45±11	<0.001	53±12	52±13	0.362
**Fasting glucose (mg/dL)**	103±26	114±40	<0.001	99±23	104±25	<0.001
**HOMA-IR**	2.46±1.61	3.59±2.48	<0.001	2.51±2.59	3.21±2.14	<0.001
**Ferritin (ng/mL)**	138±192	149±136	0.389	57±58	71±57	<0.001
**Vitamin D (ng/mL)**	21.1±7.3	18.5±6.4	<0.001	18.1±6.9	16.6±6.7	<0.001
**ALP (IU/L)**	236±70	247±78	0.034	230±76	247±79	<0.001
**i-PTH (pg/mL)**	65.1±25.2	74.7±36.3	<0.001	67.9±30.3	76.8±36.6	<0.001
**eGFR (mL/min/1.73 m^2^)**	88.0±14.1	77.7±18.7	<0.001	92.4±14.6	88.8±17.1	<0.001
**CKD stage**						
** Normal and Stage 1**	2347 (48.6)	62 (25.9)	<0.001	3813(62.0)	215(52.8)	<0.001
** Stage 2**	2298 (47.6)	136 (56.9)		2148(34.9)	165(40.5)	
** Stages 3–5**	186 (3.9)	41 (17.2)		187(3.0)	27(6.6)	

BMI, body mass index; WBC, white blood count; HOMA-IR, homeostasis model assessment estimate of insulin resistance; ALP, alkaline phosphatase; i-PTH, intact-parathyroid hormone; eGFR, estimated glomerular filtration rate; CKD, chronic kidney disease.

**Fig 1:**
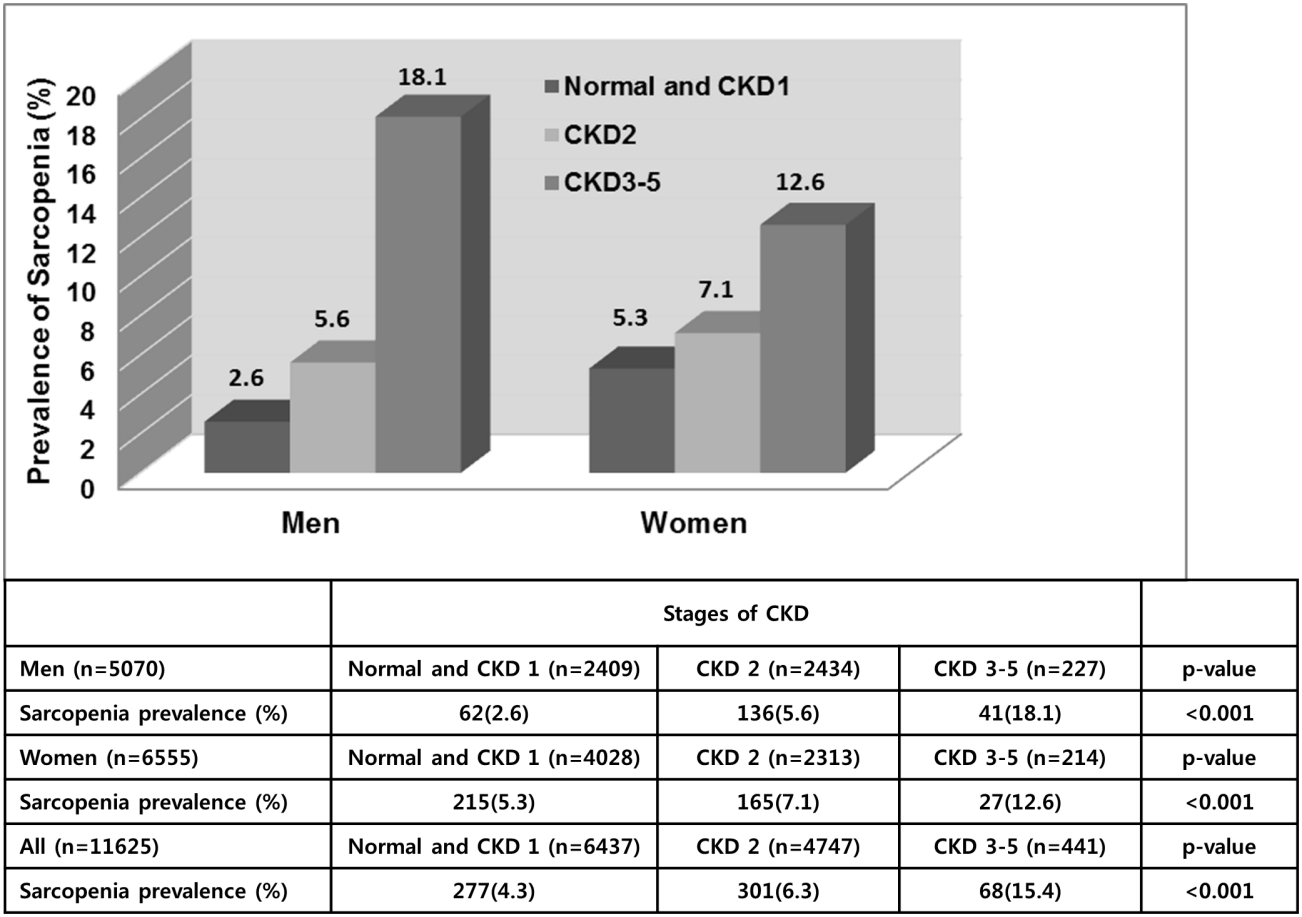
Prevalence of sarcopenia according to chronic kidney disease stage.

**Fig 2:**
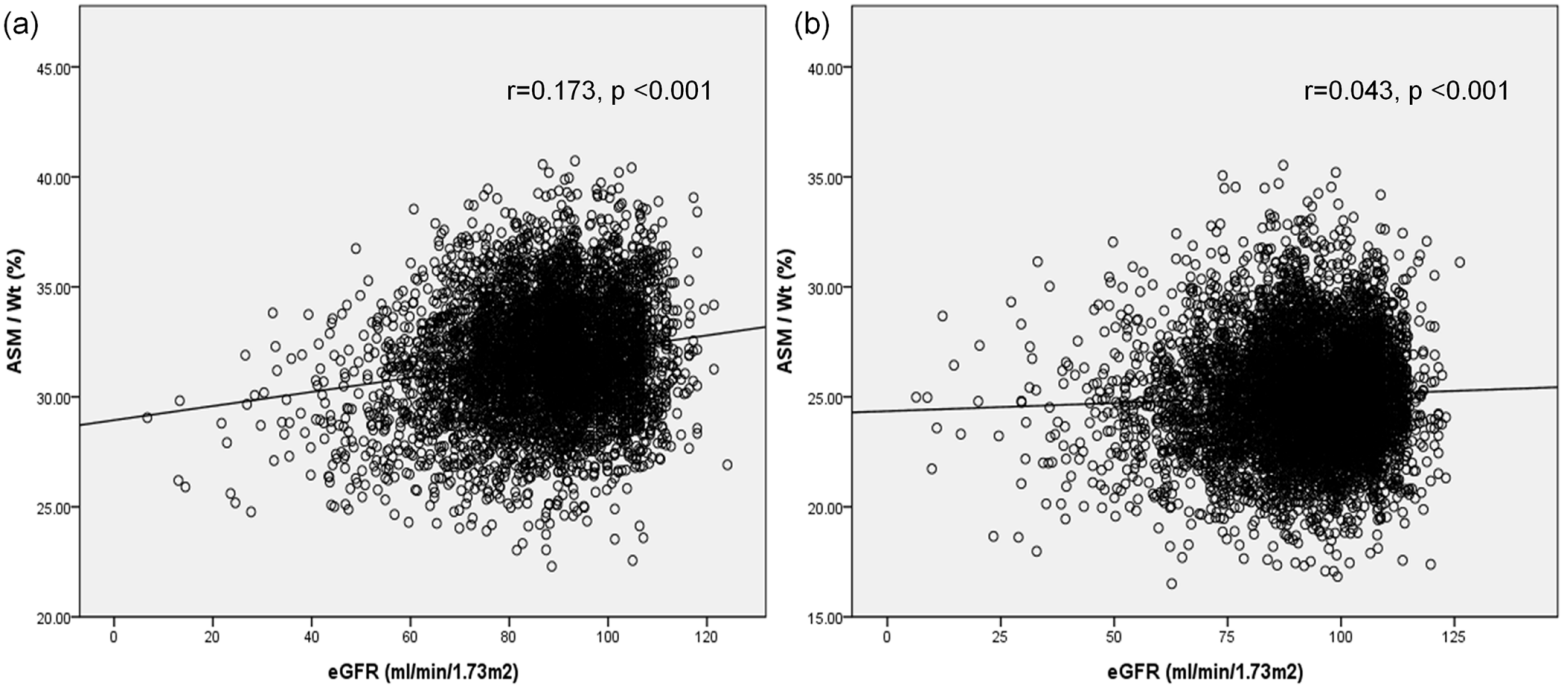
Correlation curve between glomerular filtration rate (GFR) and appendicular skeletal muscle mass / weight (ASM/Wt) in (a) males and (b) females.

The eGFR level was lower in the sarcopenia group than in the group without sarcopenia, and the proportions of moderately and severely decreased GFR were higher. As the stage of CKD increased, the prevalence of sarcopenia increased (normal and CKD 1, 2, and 3–5: 2.6%, 5.6%, and 18.1% in men, and 5.3%, 7.1%, and 12.6% in women, respectively; p<0.001; Fig 1). In addition, the correlation between GFR and ASM/Wt was weak but statistically significant in both men and women (r = 0.173 in men, r = 0.043 in women, p < 0.001; Fig 2).

### Multivariate logistic regression analyses for sarcopenia

Logistic regression analyses were conducted to investigate the association between stage of CKD and sarcopenia (Table 2). Compared to the normal and CKD 1 group, the unadjusted OR (95% CI) for sarcopenia in the CKD 3–5 group was 8.34 (5.47–12.72) in men and 2.56 (1.67–3.92) in women. The OR for sarcopenia was attenuated but remained statistically significant in men after adjusting for age and height [OR: 4.22 (2.65–6.74), p < 0.001; Model 1] but was not significant in women. In Model 3, after adjusting for age, BMI, caloric intake, log(physical activity), vitamin D, and log(HOMA-IR), the OR (95% CI) for sarcopenia with respect to CKD 3–5 was 1.93 (1.02–3.68, p = 0.035) in men. There was no statistically significant association between sarcopenia and this stage of CKD in women (CKD 3–5: OR 0.69 (0.34–1.39, p = 0.295).

**Table 2:** Odds ratios of sarcopenia according to stage of chronic kidney disease.

	Normal and CKD 1	CKD 2	p-value	CKD 3–5	p-value
**Men**	**(*n* = 2409)**	**(*n* = 2434)**		**(*n* = 227)**	
** Unadjusted**	1	2.24 (1.65–3.04)	<0.001	8.34 (5.47–12.72)	<0.001
** Model 1**	1	1.51 (1.09–2.11)	0.01	4.22 (2.65–6.74)	<0.001
** Model 2**	1	1.22 (0.86–1.72)	0.397	2.74 (1.67–4.50)	0.001
** Model 3**	1	1.17 (0.77–1.78)	0.362	1.93 (1.02–3.68)	0.035
**Women**	**(*n* = 4028)**	**(*n* = 2313)**		**(*n* = 214)**	
** Unadjusted**	1	1.36 (1.11–1.68)	0.004	2.56 (1.67–3.92)	<0.001
** Model 1**	1	0.91 (0.71–1.17)	0.155	1.20 (0.75–1.93)	0.899
** Model 2**	1	0.83 (0.64–1.08)	0.061	1.02 (0.61–1.71)	0.561
** Model 3**	1	0.78(0.57–1.07)	0.126	0.69 (0.34–1.39)	0.295

Model 1: adjusted for age and height.

Model 2: adjusted for age and body mass index.

Model 3: adjusted for age, body mass index, caloric intake, log(physical activity), vitamin D, and log(HOMA-IR).

The stages of chronic kidney disease (CKD) were divided according to estimated glomerular filtration rate: Normal and CKD 1 (≥ 90 mL/min/1.73 m^2^), CKD 2 (60–89.9 mL/min/1.73 m^2^), and CKD 3–5 (<60 mL/min/1.73 m^2^).

## Discussion

Sarcopenia is a syndrome characterized by progressive loss of muscle mass and strength [3]. Generally, metabolic disturbances such as anorexia, hypermetabolism, and chronic inflammation are well known in end-stage renal disease patients and can be explained as protein-energy wasting [13]. Sarcopenia is a feature of protein-energy wasting and aging in dialysis patients. Various studies have reported reduced exercise capacity and loss of skeletal muscle mass (sarcopenia) and subsequent loss of muscle strength in dialysis patients compared to age-matched controls [14,15]. However, it is not well known whether sarcopenia is associated with stage of kidney function in a community-based population in Korea. We found that the prevalence of sarcopenia was higher in patients with even only mildly reduced kidney function, and the stage of CKD was significantly associated with an increased prevalence of sarcopenia in men but not in women.

Foley et al. [9], using data from the Third National Health and Nutrition Examination Survey, reported that 4.5% of the study population had sarcopenia. According to stage of CKD, the prevalences of sarcopenia were 3.8%, 5.3%, and 9.4% in CKD 1, 2, and 3–5, respectively. Our results are consistent with that study, although we found slightly higher prevalences (CKD 1, 2, and 3–5: 4.3%, 6.3%, and 15.4%, respectively); this discrepancy may be the result of differences in the definition of sarcopenia, the measurement of muscle mass, and/or ethnicity. In Foley’s study, 76.9% of the study population was Caucasian, and skeletal muscle mass was calculated using the BIA equation developed by Janssen et al. [16].

Recently, Kim JE et al. reported that early-stage chronic kidney disease, insulin resistance, and osteoporosis were risk factors for sarcopenia in an aged Korean population [17]. Their study evaluated the relationship between sarcopenia and CKD, similar to the present study. However, there are several differences between the two studies, including study duration, definition of sarcopenia, and age of enrolled patients. Kim’s study enrolled patients older than 65 years of age between 2008–2009, and sarcopenia was defined as ASM divided by height^2^ with less than one standard deviation below the mean for a younger reference group. However, our study enrolled patients 40 years of age and older, had a longer duration study period (2008–2011), and defined sarcopenia as less than two standard deviations of ASM divided by weight (ASM/Wt).

The European Working Group on Sarcopenia in Older People (EWGSOP) published a report titled “Sarcopenia: European consensus on definition and diagnosis” in 2010. In the report, sarcopenia was defined as appendicular skeletal muscle mass/height^2^ for 2SD below the mean of young adults. However, in a Korean population, this criterion tends to underestimate the prevalence of sarcopenia, especially in women. Kim YS et al. reported that the prevalence of sarcopenia in a Korean elderly population was 0.1% for women when using a height-adjusted definition but 11.8% when using a weight-adjusted definition [18]. Similarly, other researchers defined sarcopenia in Koreans using a weight-adjusted definition rather than a height-adjusted definition [19–21]. In the present study, we defined sarcopenia using appendicular skeletal muscle mass/weight.

CKD stage was not associated with sarcopenia in women in the present study. Because the prevalence of sarcopenia in women with normal renal function was more than two times higher than that in men (Fig 1), it is possible that the effect of decreased renal function on sarcopenia could be weaker in women than in men. Another possibility is related to Korean-specific gender role attitudes, especially with regard to the division of household labor. Wives participate in the majority of household labor, and this gender segregation is typical in middle-aged Korean families. Therefore, elderly women may retain their muscle mass to a greater extent than elderly men, in spite of their health status.

In addition, the prevalence of sarcopenia in men increases sharply in stages 3–5 CKD. Consistent with our findings, Kopple et al. [22] reported that, in men but not women, the slope of the relationship was higher in the lower GFR group for BMI and arm muscle area, based on data from the Modification of Diet in Renal Disease study. They explained that the reduced protein and energy intakes of patients with CKD may contribute to the decline in anthropometric and biochemical nutritional parameters. Another study showed similar gender-specific differences in sarcopenia [23].

Physical activity was decreased in sarcopenic subjects compared to healthy subjects irrespective of gender. Physical activity may have protective benefits against the loss of muscle mass. Alternatively, low muscle mass may reduce one’s ability to participate in physical activities. Several previous reports found that sarcopenia was negatively associated with walking ability [24], with gender differences showing stronger associations in men than in women [21]. A longitudinal study conducted in Japan over five years showed that the risk of developing sarcopenia was substantially lower in elderly people who took at least 7,000 to 8,000 steps per day or exercised for at least 15 to 20 minutes per day at an intensity greater than three METs [25]. However, that was a cross-sectional study; therefore, further study is needed to determine a causal relationship between physical activity and sarcopenia.

In our study, the population with sarcopenia weighed more and had a higher BMI than the non-sarcopenia group in both men and women (weight and BMI: in men, 72.6±10.8 vs. 67.4±9.9 kg and 26.5±3.1 vs. 23.8±2.9 kg/m^2^; in women, 63.3±9.6 vs. 57.1±8.5 kg and 27.4±3.6 vs. 23.8±3.1 kg/m^2^). However, the sarcopenia group had lower total muscle mass but higher total fat mass. These findings indicate that the sarcopenia group had features of sarcopenic obesity: increased weight and fat levels but decreased muscle. Biolo et al. [26] explained that there were different metabolic trajectories for muscle loss versus fat changes in aging and chronic diseases. In some patients, inflammation induces anorexia and fat loss in combination with sarcopenia. In others, appetite is maintained despite activation of systemic inflammation, leading to sarcopenia with normal or increased BMI. Inactivity contributes to sarcopenia and increased fat tissue in aging and disease. Inactivity, inflammation, age-related factors, anorexia, and unbalanced nutrition affect changes in skeletal muscle and fat mass and may induce sarcopenic obesity and sarcopenia with normal/increased BMI [27]. The relationships among inactivity, inflammation, aging, anorexia, and CKD are well known [28,29]. The factors listed above also influence kidney function.

In addition, the sarcopenic group had higher levels of fasting glucose, HOMA-IR, ferritin, and intact parathyroid hormone (i-PTH) but lower levels of vitamin D than the non-sarcopenic group. Patients with CKD commonly exhibit increased insulin resistance and i-PTH but decreased vitamin D levels. Our results indicate similar laboratory findings between sarcopenia and CKD patients. Although it was not possible to investigate the effects of CKD on sarcopenia, or vice versa, due to the cross-sectional nature of the study, patients with sarcopenia and CKD may share common risk factors such as insulin resistance and low vitamin D level.

This study has several limitations. First, the cross-sectional nature of the study made it impossible to assess any cause-and-effect relationship. Further prospective research is warranted to better assess any causal relationship(s) between kidney function and sarcopenia. Second, eGFR in the study was calculated using the CKD-EPI equation, which involves age, gender, and creatinine level. However, serum creatinine level is affected by muscle mass [30] because creatinine is derived from the breakdown of muscle. If the eGFR equation using cystatin-C rather than creatinine had been used, the result would likely have been more accurate. Third, although the European Working Group on Sarcopenia in Older People developed a practical clinical definition and diagnostic criteria for age-related sarcopenia using the presence of both low muscle mass and low muscle function [3], most previous studies have used the term ‘sarcopenia’ to indicate only low muscle mass. In this study, we used a definition of sarcopenia in which ASM was calculated as a percentage of body weight because this measure showed a closer association with metabolic parameters than when defined by ASM/height^2^ [31]. Finally, because of the nature of the survey questionnaires, we could not exclude effects of information bias.

In conclusion, sarcopenia was common in community-based adults with CKD in a representative sample of elderly Koreans. The prevalence of sarcopenia was higher in patients with even mildly reduced kidney function. Stage of CKD was associated with an increased prevalence of sarcopenia in men but not women. Thus, we should evaluate the risk of sarcopenia and seek to prevent it, even when treating patients with early CKD.
